# Bias and discrimination in healthcare through the eyes of future professionals: a qualitative study of student observations

**DOI:** 10.1186/s12909-026-09461-8

**Published:** 2026-05-18

**Authors:** Ursula Meidert, Laura Candolfi, Anna Gazzotto, Frank Wieber, Andreas Gerber-Grote

**Affiliations:** 1 School of Health Sciences, Zurich University of Applied Sciences, Katharina-Sulzer-Platz 9, Winterthur, 8400 Switzerland; 2 School of Applied Psychology, Zurich University of Applied Sciences, Pfingstweidstrasse 96, Zurich, 8005 Switzerland; 3 Department of Psychology, University of Konstanz, Universitätsstrasse 10, Konstanz, 78457 Germany

**Keywords:** Healthcare disparities, Bias, Discrimination, Health equity, Vulnerable populations, Minority groups, Health profession students, Professional socialisation, Clinical training

## Abstract

**Background:**

Bias and discrimination by healthcare professionals threaten quality of care and disproportionately affect minorities and vulnerable individuals, thereby contradicting ethical standards of equal treatment. During professional socialisation, health profession students acquire clinical skills and internalise norms and behaviours by observing healthcare professionals. This study explored biased and discriminatory behaviour towards patients in clinical training environments, as observed by health profession students.

**Methods:**

Within a cross-sectional online study (*N* = 2176 students) on discrimination in healthcare at ZHAW Zurich University of Applied Sciences, School of Health Sciences, Switzerland, in June and September 2024, students’ experiences of witnessing biased and discriminatory patient care were assessed. Free-text responses were analysed qualitatively using deductive and inductive coding according to Kuckartz.

**Results:**

Out of 2176 eligible BSc and MSc healthcare students, 422 answered the question whether they had observed biased or discriminatory care of patients by healthcare staff during clinical training (response rate 19.4%). Of those, 210 (49.8%) reported witnessing such incidents and 182 specified them in free-texts. The analysis revealed a wide range of biased and discriminatory actions, most frequently withholding care, support, or medication (*n* = 58), verbal discrimination (*n* = 57), and ignoring patients (*n* = 14). Nine accounts included physical mistreatment. Reported reasons for discrimination were ethnicity/foreign origin (*n* = 48), diagnosis (*n* = 35; particularly mental health), socio-economic status (*n* = 9), gender (*n* = 9), age (*n* = 7), and disability (*n* = 7). Where specified, biased and discriminatory behaviour was attributed to care-team members (*n* = 45) or supervising professionals (*n* = 14). Reported consequences for patients included prolonged pain, fear, and insecurity.

**Conclusions:**

Healthcare students witnessed biased or discriminatory behaviour in clinical settings. This is concerning for patients, but also for students, who may internalise inequitable clinical norms during professional role formation, potentially perpetuating discrimination and contributing to health inequities. These results emphasise the need to implement educational measures (e.g., structured reflection, cultural competence, unconscious bias training), and systemic and organisational approaches to foster learning environments based on equity and respect. This includes supportive institutional frameworks, reporting systems for observed or experienced discrimination, and clinical learning environments in which equitable practice is actively modelled and reinforced.

## Background

The professional ethos of healthcare providers is grounded in the principle of treating all individuals equally, regardless of their gender, religion, nationality or race or any other physical and mental characteristics [1, 2]. However, provider biases are a well-documented contributor to health disparities [3–5]. Discrimination against patients, whether rooted in unconscious or conscious bias, poses a significant challenge to the quality of care [6–8].

The term *bias* refers to a tendency to favour one group over another, either consciously or unconsciously [9, 10]. While conscious bias and attitudes can be regulated, unconscious attitudes cannot [11]. Closely related are the concepts of *stereotype* and *prejudice* [12]. Stereotypes are typically negative, oversimplified attributes that individuals learn to associate with specific cultural or social groups and their members [9, 13, 14]. Prejudice, by contrast, denotes the outward expression or negative attitudes towards groups or individuals [15]. *Discrimination* is the resulting behavioural action towards individuals based on stereotypes and prejudice about the group they are associated with. In healthcare, stereotypes and prejudice may lead to professionals consciously or unconsciously linking physical or social characteristics of a patient with a particular group and inferring expectations about traits or behaviours [7, 11, 12]. This can result in differential treatment of patients. While we acknowledge that bias and discrimination are rooted in historical developments and are shaped by structural factors such as professional hierarchies, this paper focuses specifically on whether and how healthcare students observe and describe bias and discrimination, as part of a larger study in which participants evaluated vignettes depicting patients with different characteristics [16].

Systematic reviews have identified a wide range of drivers for stereotyping, prejudice, or discrimination in healthcare. Common attributes include age, gender, sexual orientation, socio-economic status, migration background, ethnicity or race, body morphology, disability, and type of diagnosis (e.g., mental illness) [11, 17, 18]. These biases influence communication between healthcare professionals and patients, including the number of questions asked, conversational dominance during consultations [19], communication quality [20], and provision of care. Documented consequences or effects include disparities in pain medication prescription [21], diagnostic procedures [22, 23], consultation or treatment delays [24, 25], consultation length, clinical decision-making [26], specialist referrals [22], and under- or over-treatment [27]. Moreover, situational factors such as fatigue, time pressure, limited clinical information, and cognitive overload can impair rational and deliberative decision-making, potentially making individuals more susceptible to heuristics and unconscious stereotypes [28]. These conditions are particularly relevant in healthcare settings, where high workload, time pressure, and cognitive burden are commonplace.

Patients seeking care are frequently in conditions of acute need and dependency on healthcare providers, including reliance on nursing care and information asymmetry between themselves and healthcare professionals [29]. This makes it particularly concerning when the professional responsibility entrusted to providers is undermined by biased attitudes or behaviours, leading to unequal care. Discriminatory encounters may undermine trust in care, discourage further care-seeking [30–32], and affect not only the patients’ own health but may also affect families when caregivers postpone or forego care for dependants, including children. Expectations of discrimination may also shape subsequent patient-provider interaction, for example by shaping communication patterns such as patients’ verbal engagement or talk time during clinical encounters [33].

Consistent findings from international literature indicate that patients from minority or historically marginalised groups often report lower levels of satisfaction with medical or nursing care compared with majority populations [29]. In Switzerland, a recent national study indicates that patients in disadvantaged socio-economic conditions tend to rate the quality of care lower overall. This pattern includes younger adults (18–34 years), single-parent households, first-generation migrants, and individuals with limited financial resources [34]. A study from the French-speaking region of Switzerland reports that 11.1% of recently discharged hospital patients reported they had experienced at least one discriminatory encounter [35]. It remains methodologically challenging to fully disentangle general differences in patient satisfaction from concrete instances of discriminatory treatment. Patient-reported satisfaction may be influenced by factors such as language competence or origin [29], which do not necessarily reflect discriminatory behaviours by staff. Nevertheless, patterns consistently highlight inequity affecting vulnerable groups [36]. Patient experiences provide indispensable insights into the quality of care, as they reflect how individuals perceive, interpret, and are affected by clinical encounters. Healthcare students offer a complementary perspective to patient reports. They are shaped by socialisation processes and institutional norms. During their internships they are not always directly involved in the encounter they observe. This relative distance may allow them to notice both explicit and subtle forms of behaviour from health professionals that individual patients might not contextualise within a broader pattern of care. Alongside patient accounts, these observations can contribute to a more comprehensive understanding of how inequities manifest in clinical practice. Extensive research has documented patients’ experiences of discrimination in healthcare and its consequences for trust, communication, and health outcomes [35]. The present study does not seek to replace this perspective, but rather to complement it by examining how discriminatory practices are observed and interpreted by healthcare students within clinical environments. While prior studies have documented the prevalence and consequences of discrimination in healthcare [35], less is known about how healthcare students perceive, classify and internalise such behaviour during clinical training, how these observations shape professional socialisation, and how they might inform educational interventions. This perspective is particularly important because students learn professional norms not only through formal curricula but also through direct observation of clinical role models. This includes the hidden curriculum, referring to implicit norms embedded in organisational structures and workplace culture [37]. Haidet and Stein [38] and Gaufberg et al. [39], have shown that hidden curricula influence professional development through interpersonal interactions, role modelling, culture, and clinical hierarchies, both positive and adverse examples. Other studies indicate that students often experience strong feelings of powerlessness when they observe a contradiction between the patient-centred care they were taught and the behaviours they witness in practice [40].

Existing research has largely focused on patient reports or self-assessments by healthcare professionals. Fewer studies capture the learner viewpoint directly [40–42], despite students occupying a dual role as learners and semi-independent observers. Our study addresses this gap by analysing students’ observations of bias and discriminatory behaviour in clinical settings, providing insight into practices that may otherwise remain hidden and highlighting areas where educational strategies could be strengthened to promote equitable care. By focusing on the Swiss clinical-training context, this work contributes to the literature on health inequalities and underscores the relevance of the clinical learning environment in shaping students’ professional socialisation.

## Methods

### Data collection

Data were collected through a cross-sectional online survey targeting healthcare students. The questionnaire comprised several sections, including items of explicit attitudes towards different groups of people, as well as vignettes designed to capture unconscious bias. This article focuses on a single-choice question of whether students had observed biased or unequal behaviour by healthcare professionals. This item functioned as a filter question, determining whether respondents were invited to provide further qualitative data. The wording of the question was *“Have you ever observed other healthcare professionals treating patients/clients unequally*,* in a demeaning or discriminatory manner?”* (English translation of the German original: “Haben Sie schon einmal beobachtet, wie andere Gesundheitsfachpersonen Patient: innen / Klient: innen ungleich, abwertend oder diskriminierend behandelt haben?*”*) The single-choice question contained the answer options: “yes”, “no”, and “I’m not sure”. Only respondents who selected “yes” were subsequently asked to describe the incident briefly using an open response format: *“Please briefly describe the situation and how it ended”.* No additional prompts were provided regarding the professional role of the persons involved, the timing, or the clinical context. The invitation to report observed unequal treatment or discriminatory behaviour was intentionally kept brief and minimally structured. This served to limit respondent burden and reduce the risk of survey attrition. However, as a side effect, responses varied in level of detail and completeness.

We used a qualitative, open-ended methodological approach for the free text question. Rather than presenting predefined categories of possible observed actions and assumed reasons, students were invited to share personally witnessed experiences. This design aimed to elicit authentic accounts from clinical practice, offering deeper insights into subtle, context-specific manifestations of biased or discriminatory behaviour that structured questionnaires may not fully capture.

Additionally, students provided information on gender identity, age, nationality, sexual orientation, study programme and semester. They were also asked about professional work experience in healthcare and, if applicable, its duration, as well as the extent to which they currently identify with their (future) profession. Furthermore, students reported on prior diversity or unconscious bias training, and their personal attitude towards equitable patient care. Further details on the development, structure, and additional components of the questionnaire are reported elsewhere [16].

### Participants

All students enrolled in a bachelor’s (BSc) and master’s (MSc) programmes in nursing, physiotherapy, occupational therapy, midwifery, and health promotion and prevention were recruited from ZHAW Zurich University of Applied Sciences, School of Health Sciences in Winterthur, Switzerland. Nursing and midwifery students undertake their first internship in the second semester, whereas students in other allied health programmes begin placements in the fourth semester. Many students may have acquired professional experience in healthcare prior to entering the bachelor’s programme, and a substantial number work in clinical settings alongside their studies. This reflects the multi-level structure of the Swiss vocational educational system, which enables progressive competency development through successive qualifications.

A total of 2176 eligible BSc and MSc students were invited via their official university email address, which is routinely used by the institution for study-related communication, including invitations to participate in research, in June 2024 (semesters 2, 4 and 6) and September 2024 (first semester only). Reminders were sent two weeks after the initial invitation. Participation was voluntary, and informed consent was obtained prior to participation. Recruitment was supported through the university’s internal homepage, flyers distributed at the school, and a raffle of three gift vouchers of CHF 150 (Euro 160) each for participating students.

### Dataset and tools

The survey was administered using Unipark© (Tivian©). After data collection, all responses were exported and anonymised to create a dataset focused on observed instances of biased or discriminatory treatment, ensuring that no participants could be identified at any stage of the analysis. The data were then imported into MAXQDA© for analysis.

### Analysis

Participant characteristics and response frequencies were calculated using descriptive statistics and Chi² tests in Stata^®^. Qualitative analysis of the open-ended responses followed Kuckartz’s [43] content-analysis approach, which combines a structured yet flexible framework development with both deductive and inductive category refinement.

Initial analytic dimensions and categories were informed by a comprehensive literature review on unconscious bias [17], which synthesised conceptual and empirical work on manifestations of bias in healthcare professionals. This review was used to develop the initial coding framework. All codes and subcodes, with illustrative examples and frequency counts, are presented in Table 2. The codes were applied and refined iteratively throughout the analysis. Responses were systematically coded, and additional categories were added through open coding when novel aspects emerged. Within each analytic dimension, categories were defined to capture distinct aspects of responses; individual answers could receive multiple codes across and within analytic dimensions and categories when clearly applicable (e.g., bias attributed to both gender and skin colour). Each response was coded across several analytic dimensions including biased or discriminatory behaviour, perceived underlying ground, perceived actors involved and perceived consequences. This iterative process allowed for a nuanced understanding of how students encountered and interpreted biased or discriminatory behaviour in clinical settings. The qualitative analysis was conducted by an interdisciplinary research team with academic backgrounds in psychology (*n* = 2) and health sociology (*n* = 1). All coders had prior experience in qualitative data analysis and were familiar with the literature on bias and discrimination.

To enhance credibility and reliability, the dataset was independently coded by two researchers, following the principle of investigator triangulation outlined by Flick [44], whereby multiple researchers analyse the same data to minimize individual bias and enhance the robustness of interpretation. Discrepancies between coders were resolved through team discussions addressing how researcher background and experiences may influence interpretation, ensuring intersubjective comprehensibility and transparency.

The final analytic dimensions and categories were reviewed for internal consistency and conceptual distinctiveness. Results were presented both descriptively and visually, with attention to transparency, systematic reporting, and accountability of analytic decisions, in line with established principles of qualitative reporting, including the Standards for Reporting Qualitative Research (SRQR) [45].

### Ethical considerations 

Ethical approval for the study was obtained by the Ethics Review Board of the Zurich University of Applied Sciences (No. EA-ZHAW 2024-008-G).

## Results

### Participants characteristics

A total of 470 out of 2,176 students participated in the online survey (overall response rate: 21.6%). Of these, 422 respondents answered the single-choice question of whether they had observed biased or discriminatory treatment of patients by healthcare staff during their clinical training and were therefore included in the present analysis (item response rate: 89.8%, 19.4% of the invited sample). Response options for this question were ”yes”, “no”, and “I don’t know/I’m not sure”. Detailed characteristics of these 422 participants included are presented in Table 1.

**Table 1:** Respondent characteristics (*n* = 422)

Characteristics	% (n)
Gender identity	
Female	91.5% (386)
Male	7.6% (32)
Non-binary or diverse	0.7% (3)
Prefer not to respond	0.2% (1)
Age	
≤20	13.7% (58)
21-25	53.6% (226)
26-30	18.5% (78)
31-35	6.9% (29)
≥36	7.4% (31)
Nationality	
Swiss	75.4% (318)
Other nationality	8.5% (36)
Dual citizenship (Swiss and other)	16.1% (68)
Study level	
Bachelor	88.2% (372)
Master	11.9% (50)
Study programme	
Physiotherapy	25.8% (109)
Nursing	23.5% (99)
Occupational therapy	19.0% (80)
Health promotion and prevention	16.6% (70)
Midwifery	15.2% (64)
Work experience	
None	2.8% (12)
Less than 6 months	18.7% (79)
6 months to 1 year	22.8% (96)
1 year to 2 years	11.1% (47)
More than 2 years	44.6% (188)

Please note that due to rounding, percentages may not add up to exactly 100%

### Witnessing biased behaviour

Of the 422 students who responded to the question, 210 (49.8%; BSc students *n* = 177; MSc students *n* = 33) reported having witnessed biased or discriminatory behaviour by healthcare staff, 88 (20.9%; BSc students *n* = 83; MSc students *n* = 5) reported none, and 124 (29.4%; BSc students *n* = 112; MSc students *n* = 12) indicated uncertainty as to whether the situations they observed constituted biased behaviour or discrimination.

The distribution of participants did not differ across study programmes. Master students reported witnessing such behaviour significantly more often than Bachelor students (66% vs. 47.6%) and were also slightly more likely to report uncertainty in their observations (30.1% vs. 24.0%; χ² *p* < 0.05).

### Content analysis

Of the 210 students who had witnessed biased or discriminatory behaviour, 182 provided a brief description of the observed situation. Qualitative analysis was conducted on these 182 free-text responses. The analytic dimensions and categories are summarised in Table 2.

**Table 2:** Analytic dimensions and categories for coding with examples and number of observations

Construct	Definition	Sample quote	N
**Perceived nature of bias or discrimination**			
Ignoring	Patient or patients’ needs are ignored	“A team colleague discriminated against patients based on their ethnicity or ignored them altogether. […]” No. 268	14
Perceived inadequate or withheld care	Patient is recognised but does not receive treatment or insufficient or sub-standard treatment	“Overweight patients, treatment was shorter.” No. 169	58
Verbal	Patient is spoken about in a derogatory manner either directly to the patient or in patient’s absence	“A male nurse once insulted a female patient, and she told me that she was afraid of him.” No. 311	57
Non-verbal	Disparaging gestures are made, such as rolling the eyes, shaking the head, or grimacing	“During an ultrasound in a prenatal check-up, the resident visibly rolled her eyes in annoyance whenever the non-native speaking couple didn’t understand one of her questions.” No. 15	1
Physical mistreatment	Patient is physically mistreated or roughly handled	“[…] When I did an observation day in the emergency department, I witnessed the nursing staff treating a dying man very roughly.” No.80	9
Undefined	Action/statement is unclear	“Discussions at the nurses' station.” No. 33	63
**Perceived reason for bias or discrimination**			
Gender	Discrimination is based on the patient's sex/gender	“Yes, I have seen how women […] are more often than men judged as hysterical or overreacting, and it is assumed that they are exaggerating their symptoms.” No. 155	9
Socio-economic status	Discrimination is based on the socio-economic status, education, or living circumstances	“People with low socioeconomic status and substance use disorders are stigmatised in psychiatry.” No. 402	9
Diagnosis	Discrimination is based on the patient's diagnosis	“A registered nurse refused to treat a patient with heroin dependence in the hospital.” No. 430	35
Body morphology	Discrimination is based on the patient's overweight, obesity, or other bodily features	“Overweight patients, treatment was shorter.” No. 169	2
Ethnicity, origin orskin colour	Discrimination is based on the patient's origin, race, or skin colour	“Foreigners had to wait longer than Swiss patients.” No. 466	48
Age	Discrimination is based on the patient's age	“Made derogatory remarks about fat or elderly people during breaks and then treated them only half-heartedly.” No. 249.	8
Sexual orientation	Discrimination is based on the patient's sexual orientation	“Derogatory comments based on education, origin, sexuality, illness; more or less empathy for the same situation; ignoring needs due to prejudices, etc.” No 187	3
Ableism	Discrimination is based on the patient's disability	“A doctor treated a patient with severe physical and mental impairment much more roughly than the nursing staff and physiotherapists during the ward round.” No. 287	7
Religion	Discrimination is based on the religious community the patient belongs to	“Belief, religion, gender, origin, age, diagnosis”. No.19	1
Undefined	Reason for the discrimination is not stated or unclear		90
**Perceived acting person**			
Team member	Acting person is a team member / a trained person	“[…] The nursing staff behaved in a derogatory, unsympathetic, provocative, and unfriendly manner towards the patients. ” No. 475	45
Supervisor or superior	Acting person is a superior, superior or otherwise in a higher position in the institution	“When I worked on a dementia care unit, my supervisor made extremely negative remarks about certain patients.” No.126	14
Student or intern	Acting person is equally a student or an intern		0
Undefined	It is unclear what function the acting person has	“Derogatory remarks”. No 61	124
**Perceived consequences**			
Physical consequences	Physical consequences of discrimination for patient	“Painkillers not administered to a foreign patient because it was interpreted that the patient was malingering, it ended up that the patient needed a high dose of opiates and was almost sedated.” No. 108	10
Psychological consequences	Psychological or emotional consequences of discrimination for patient	“A male nurse once insulted a female patient, and she told me that she was afraid of him.” No. 311	11
Undefined	No consequences are mentioned/consequences are unclear	“Pain wasn’t recognised; credibility was questioned. Sentences such as he/she just doesn't want to work etc. are often heard.” No. 186	161

Please note that the reported frequencies exceed the total number of free-text descriptions (n = 182) because individual responses could include and be coded for more than one aspect of perceived biased or discriminatory behaviour, perceived reason for bias or discrimination, perceived acting person, and perceived consequences

The responses indicated that biased or discriminatory behaviour was not limited to isolated incidents but extended to various forms of interaction, actors, and patient characteristics. The data suggest that such behaviour occurred both in direct patient contact and within the team context, ranging from subtle forms of exclusion to more explicit mistreatment. However, the level of detail varied considerably from one response to another, making a more precise interpretation difficult.

Some responses were very brief and provided little contextual information about the situation perceived as biased or discriminatory (e.g., “several times” or “stigmatisation”). As a result, these responses were coded as “unclear” regarding the nature and perceived reason of biased or discriminatory behaviour, the actors involved, or the perceived consequences of the incident. This code was assigned because, although the students reported experiencing an event, the descriptions were too unspecific to extract further analytic detail.

### Nature of biased or discriminatory behaviour

The analysis distinguished six subcategories of biased or discriminatory behaviour: ignoring; inadequate or withheld care; verbal, non-verbal, physical mistreatment; and “unclear”, which contained undefined cases, where students didn’t provide enough information to categorise their observation (see above). The most frequently reported form of biased or discriminatory behaviour involved perceived inadequate or withheld care or support, and sub-standard treatment. In 58 cases, students perceived situations in which appropriate care was deliberately withheld. Examples included deliberately delaying support and care, delayed or restricted access to pain medication, shortened or sub-standard treatments, or denial of care. One nursing student noted:

*“Pain medication prescription was not issued because the staff “had something else to do.” Checking it would have taken one minute. The patient had to wait ten minutes in severe pain.”* (No. 13).

Another student reported:

*“Pain medication was withheld from a foreign patient because it was interpreted that the patient was faking. This ultimately resulted in the patient requiring a high dose of opioids and becoming nearly sedated.”* (No. 108).

A commonly mentioned omission concerned patients not being appropriately informed or excluded from decision-making:

“*In cases of communication barriers*,* decisions are made over the patient’s head rather than through informed consent*.” (No. 9).

Verbal discrimination was the second most frequently observed form of discriminatory behaviour (57 mentioned). This included rude or impolite or derogatory speech, stigmatising statements, and discriminatory remarks linked to patient nationality, sexual orientation, or certain diagnosis (e.g., substance addiction). In 23 cases, comments were directed at patients, such as overt expressions of dislike, patronising remarks, speaking to patients as though addressing children, or belittling them. More aggressive forms included yelling, cursing, insulting, or snapping at patients, often in interactions with patients experiencing delirium, dementia, (cognitive) disabilities, or language barriers:

*“As a student job*,* I’m a sitting guard in hospital and have often experienced nurses talking derogatorily about patients. Sometimes even in the presence of the patient if they were delirious or similar.”* (No. 4).

In 14 cases, derogatory comments occurred away from patients (e.g. in the staff rooms or team meetings). In 20 cases, it remained unclear whether comments were made in patients’ presence or could be overheard, as descriptions were inconclusive, for example:

*“Derogatory*,* stigmatising*,* and discriminatory statements regarding nationality*,* sexual orientation*,* and low socioeconomic status.”* (No. 133).

Patients of foreign nationality were particularly affected by verbal discrimination, with some derogatory remarks made in their presence including complaining to the patient that he/she did not speak German (the language spoken where students have their internships).

Non-verbal forms of discrimination were also reported, including visible annoyance such as eye-rolling (No. 446), or unnecessary precautionary measures, including the use of overly protective clothing when treating a patient with HIV and darker skin (No. 357). These incidents were described particularly in interactions with patients who did not speak German fluently or were of foreign nationality.

In 14 responses, students described discriminatory behaviour as ignoring patients’ needs:

*“Dismissive comments based on education*,* origin*,* sexuality*,* or illness; varying levels of empathy in identical situations; ignoring patients’ needs due to prejudice*,* etc.»* (No. 187).

A small number of participants reported incidents involving physical aggression or coercion (9 cases), including rough handling during treatment or hygiene procedures, holding a patient’s mouth shut, administering care against patient will, of performing procedures (e.g., catheterisation or medication) without consent.

For 58 responses, information was too limited to identify a specific form of discrimination or to support coding within at least one analytic dimension regarding the nature of biased or discriminatory behaviour.

In summary, the reported behaviours suggest that discrimination was often embedded in routine care interactions, particularly through omissions (e.g., withholding care or information), while everyday communication practices and overtly aggressive actions were relatively rare.

### Perceived reason for biased or discriminatory behaviour

The dimension “perceived reason for biased or discriminatory treatment” included the following subcategories: gender, socio-economic status, diagnosis, body morphology, ethnicity/origin/skin colour, age, sexual orientation, ableism, religion, and undefined for comments that could not be classified. Within this dimension, the reasons most frequently reported by students were patients’ foreign origin, race or ethnicity, expressed via nationality, skin colour, or limited German language proficiency. Some students described overt racism, while others noted more subtle forms of xenophobia, including assumptions about intelligence, parental competence or questioning the patient’s credibility:

*“A team colleague discriminated against patients based on their ethnicity or ignored them altogether. She began yelling*,* and the patient was very distressed.”* (No. 268).

Racist or ethnic stereotypes were also reported to influence clinical interpretation; for example, students described instances in which foreign patients’ symptoms were trivialised. Several students referred to the so-called “Mamma Mia syndrome” or “Mediterranean syndrome”, colloquial terms implying that patients from Mediterranean countries exaggerate pain.

*“In cases involving a migration background*,* pain perception is often attributed to the patient’s nationality — for example*,* with stereotypes like the so-called “Mamma Mia syndrome” applied to Italian patients.”* (No. 240).

Other factors included patient diagnosis. Most frequently referenced were mental illness, addiction and dementia. Apart from mental illness, patients with chronic pain conditions were often not taken seriously or otherwise treated unprofessionally. Additional accounts mentioned patients with HIV, and incontinence.

Other ascribed characteristics included gender (*n* = 9), socioeconomic status (*n* = 9), age (*n* = 8), disability (*n* = 7), sexual orientation (*n* = 3), body morphology (*n* = 2), and religious affiliation (*n* = 1). Students emphasised that biased or discriminatory behaviour that they witnessed disproportionately affected patients experiencing vulnerability, including older age, insecure housing, or dependency on clinical support. Some accounts described intersecting vulnerabilities:

*“A (…) woman (60)*,* who has multiple diagnoses and therefore experiences severe cognitive*,* physical*,* and neurological impairments*,* was frequently ignored and neglected because she was unable to defend herself verbally or nonverbally.”* (No. 134).

Across the responses, the findings suggest that perceived reasons for biased or discriminatory behaviour were predominantly linked to ascribed patient characteristics, particularly those associated with social difference and vulnerability. The accounts are consistent with the interpretation that these attributions may influence not only interpersonal behaviour but also clinical interpretation, for example in the assessment of symptoms. At the same time, several responses point to intersecting vulnerabilities, indicating that discrimination may arise from the combined effect of multiple patient characteristics rather than a single factor.

### Acting person

The category “acting person” included the subcategories team member, supervisor or superior, student or intern and, undefined for answers that could not be categorised. Most biased or discriminatory behaviour was exhibited by team members, i.e., experienced healthcare professionals. In 45 cases, a team member or a trained healthcare professional was responsible, and in 14 cases, the behaviour was attributed to a supervisory or higher-ranking staff. Notably, no reports implicated fellow students or interns. In most descriptions (*n* = 124), the acting individual was not specified.

Taken together, the available data suggest that biased or discriminatory behaviour was primarily attributed to experienced healthcare professionals rather than trainees. At the same time, the large proportion of unspecified actors indicates that such behaviour was often described without clear attribution, reflecting the limited contextual detail of the responses and possibly the observational position of students within hierarchical clinical settings.

### Perceived consequences

The category “perceived consequences” included the subcategories physical consequences, psychological consequences, and “undefined” for cases where insufficient information was provided. In many responses (*n* = 161), consequences for affected patients or staff were not described or remained unclear and were therefore coded as undefined, (e.g., “*patients being handled roughly*”; No. 80). Where specified, perceived consequences included physical (*n* = 10) and emotional or psychological impacts (*n* = 11). The most commonly described physical consequences were patients enduring avoidable pain, or being “*left in wet pads”* (No. 27). Perceived psychological or emotional reactions included feelings of being intimidated, irritated, humiliated, shocked, fearful, insecure, or experiencing a disrupted sense of trust. Students also noted subsequent dissatisfaction with care, care avoidance or resistance, including patients refusing treatment, declining further communication, or foregoing care altogether:

*“The healthcare staff treated this woman so badly that she became afraid of them. […]”* (No. 495).

Some students explicitly stated that they could not assess the consequences for patients.

Consequences for the acting staff were rarely reported. The few follow-up actions described included patient’s refusal to be treated by the respective health professional, discharge of health professionals, supervisory interventions, and in one case, de-escalation training. In some cases, “nothing was done” (e.g. 495), and discriminatory behaviour continued.

Students themselves were also affected, reporting feelings of unease, irritation or being “shocked”. Others described active responses, including speaking up or intervening. One student wrote that after reporting an incident to a supervisor, she/he had to justify her/himself for doing so. One participant reported resigning due to the situation.

Overall, consequences of discriminatory behaviour were only rarely described in detail. This lack of explicit reporting may reflect both the limited depth of the responses and the difficulty for students to assess outcomes beyond the immediate situation. Where consequences were mentioned, they primarily referred to immediate physical discomfort or psychological distress, as well as disruptions in trust and care relationships. The predominance of unspecified consequences further suggests that the impact of such situations may remain insufficiently visible or is not systematically addressed in everyday clinical practice.

### Assumed causes and influencing circumstances

Several students linked discriminatory behaviour to stress, high workload, staffing shortages, time pressure, and fatigue:

*“Various situations*,* mostly associated with stress. Snapping at patients when they have an extra request or barely responding (mumbling & paraverbal derogatory).”* (No. 12).

Students also observed differences in care interactions that they associated with varying patient communication styles, including how confidently or assertively patients expressed their needs:

*“Patients who frequently and more assertively demand support often get more attention*,* while those who are quieter and do not want to burden us get less attention”.* (No. 22)

However, patients who were more assertive sometimes were also described by staff as annoying or overly demanding, which was then used to justify worse treatment. Discrimination was also observed when patients were perceived as ”immoral”, for instance:

*“[…] women who have a desired abortion are not always treated with empathy.”* (No. 122).

In summary, these accounts suggest that biased or discriminatory behaviour was often perceived as situationally embedded, particularly in contexts characterised by high workload, time pressure, and demanding interactions. At the same time, patient-related factors, such as communication style or perceived assertiveness, appeared to influence how care was delivered, indicating that both contextual and interpersonal dynamics may shape these situations.

Overall, the findings illustrate that students observed a broad spectrum of biased and discriminatory behaviours across different clinical contexts. While the data do not allow for systematic analysis of patterns or causal relationships due to their brevity and limited contextual detail, they consistently point to the presence of such behaviours in everyday clinical practice, involving different professional roles and affecting particularly vulnerable patient groups. The accounts further suggest that these situations are not always explicitly addressed and may, in some cases, go without consequences.

## Discussion

This study examined how health profession students observe, perceive and interpret biased or discriminatory behaviour towards patients in clinical practice. Approximately half of respondents reported witnessing such incidents ranging from subtle, potentially unconscious bias to more explicit forms of discrimination, with consequences for patients varying from minor to more tangible.

Consistent with prior research, discrimination was most frequently attributed to patients’ origin, ethnicity, and limited ability to communicate in German [35]. Given that Switzerland has one of the highest net migration rates among OECD countries [46], these findings are of particular concern. In such a diverse healthcare context, training in cross-cultural communication, diversity, and unconscious bias is widely considered important. However, a focus on individual training alone is insufficient to address the broader patterns of discrimination observed, which are also shaped by stereotypes, power relations, and structural conditions within healthcare settings [47]. While many healthcare institutions have formal mechanisms such as feedback loops, quality assurance processes, or patient surveys in place, our findings do not allow conclusions about the extent to which these mechanisms capture or address experiences of discrimination. Rather, the students’ accounts highlight that such experiences occur in clinical practice in Switzerland and may not always be explicitly addressed.

International findings are comparable and strengthen this interpretation. In a U.S. study, 55% of health professions students reported observing unconscious bias towards patients based on race or ethnicity, most often through reduced patient education, fewer questions, or shorter treatment sessions [41]. Similarly, an Israeli study found that 52% of medical students had witnessed biased treatment, frequently related to patients’ background, race or beliefs, often in the form of derogatory remarks [42]. While such results may reflect the prevalence of biased or discriminatory incidents, they may also suggest that students are particularly attuned to professional equity standards and therefore more sensitive to perceived deviations.

Notably, many students in our study expressed uncertainty about whether they observed constituted discrimination, reflecting the inherent challenges in identifying biased behaviour in clinical contexts. Similar themes have been reported in the literature: students may struggle to determine the severity or reportability of behaviours perceived as unprofessional and rely on peer discussions to interpret experiences of unprofessional conduct (e.g., hidden curriculum dynamics) [48]. In this sense, students’ observations can be understood as early inputs into processes of professional socialisation, in which learners are confronted with implicit norms and expectations conveyed through everyday clinical practice. However, the present study does not examine whether or how such observations are internalised into stable professional attitudes or behaviours.

Research on healthcare students’ perceptions of bias and discrimination suggests that even with training, students can find it difficult to recognise and categorise biased and discriminating behaviours in clinical settings [41]. This reflects the complexity of identifying biased and discriminating behaviour and the challenges students face in interpreting clinical situations. Although many students struggled to classify bias and discrimination with confidence, this ambiguity itself holds pedagogical relevance, underscoring the need for deliberate training in recognising and naming inequitable care. Several responses further indicated that the observed behaviours may have been unconscious on the part of acting professionals, adding to the difficulty in categorising and attributing cause.

A further observation is that no students reported witnessing biased or discriminatory behaviours by peers. Rather, reports focused on clinical staff, often in supervisory or senior positions. As students were not explicitly asked about peer behaviour, it remains unclear whether this reflects a true absence of such incidents or limitations in observation or recall. One possible interpretation is that the absence of reported peer discrimination does not necessarily mean that it did not occur but may rather reflect how hierarchical role modelling shapes students’ perceptions of what constitutes acceptable professional behaviour. This is consistent with findings by Song and Willy [49], who reported that students observed relatively little unprofessional behaviour among peers compared to licensed healthcare professionals, highlighting the influence of clinical supervisors on students’ perceptions of professional norms and/or the relative uncritical appraisal of their peers’ behaviour. These observations are noteworthy, as senior staff serve as role models during students’ professional socialisation. Although we cannot assume direct effects on learner attitudes, the number and nature of reported incidents are concerning. When such behaviour is normalised or left unchallenged, it may influence students’ perceptions of professional norms and expectations, emphasising the potential tension between taught standards and the observed clinical practice [49]. Such tensions are central to theories of professional socialisation, which emphasise how discrepancies between formal curricula and observed practice may shape learners’ understanding of acceptable professional conduct [50, 51]. As other studies have shown, such contradictions may lead to tension, powerlessness, insecurity about expectations [40], exhaustion or disengagement, and may be associated with reduced empathy [52]. Furthermore, witnessed biased behaviour not only limits students’ learning opportunities but also undermines their confidence, discourages them from seeking guidance, mentorship, or constructive feedback. These experiences ultimately affect their ability to focus, perform at their best, and develop their full potential as future healthcare professionals [49]. A systems perspective, as emphasised in frameworks by the World Health Organization [53], supports examining discriminatory encounters not only as isolated events, but as signals of a complex care system in which multiple interacting factors shape clinical behaviour and learning conditions. These findings further suggest that biased or discriminatory practices are not solely attributable to individual actors but are embedded in organisational routines, hierarchies, and institutional cultures that shape both care delivery and learning environments.

Another observation is the limited reporting of institutional or supervisory follow-up in student accounts. While students rarely mentioned consequences, accountability processes, or structured organisational responses, it is important to note that they were not explicitly asked about these aspects; they were only asked to describe “how the situation ended”. Therefore, no conclusions can be drawn regarding the presence or absence of systematic procedures [41]. Yet, promoting a speak-up culture, as emphasised by Schwappach & Richard [54], and opportunities for peer discussion may help students to contextualise such discriminatory encounters, as a study with medical students in Switzerland has shown [55].

### Implications for practice and education

The present findings highlight the need to foster a clinical learning culture in which equitable treatment is a core value, biased or discriminatory behaviour is not tolerated, and incidents are openly and constructively addressed. Such a culture benefits not only patients, who are often in vulnerable situations, but also students, who are shaped by professional norms they observe, and healthcare professionals, whose commitment to equity is reinforced. Institutions should thus strengthen accountability and actively promote health equity within both care delivery and professional training [56].

Interpretation and implementation of these findings should take into account contextual factors such as reporting structures, potential consequences of raising concerns, and power dynamics within clinical settings, which may shape students’ experiences of and responses to discriminatory situations. These contextual factors point to the importance of organisational conditions in shaping both the occurrence of discriminatory practices and students’ opportunities to respond to them.

From an educational perspective, health professions programmes should create structured opportunities for students to reflect on witnessed discriminatory encounters, for example, through guided debriefings, supervision, or ethics rounds [57, 58]. Clinical educators, who serve as powerful role models in shaping professional identity, should receive ongoing training that emphasises their responsibility to model equitable behaviour and address inequality, prejudice, and unconscious bias in everyday practice. Curricula should also integrate cultural competence and unconscious bias training, complemented by practical tools such as bystander-intervention strategies, enabling students and staff to recognise, name, and respond to discriminatory practices [41, 59]. Thereby, also contextual factors such as reporting structures, potential consequences of raising concerns, and power dynamics within clinical settings should be part of the curricula.

By embedding these elements more systematically into health professions education, institutions can not only protect patients and support learners but also contribute to a long-term transformation towards more inclusive and socially responsive healthcare.

### Strengths and limitations

A key strength of this study is the open formulation of the survey question which was designed to elicit unprompted observations of discriminatory behaviour without imposing predefined dimensions and categories. The results therefore provide valuable insights into perceptions of inequality, biased or discriminatory behaviour from the perspective of students of health professions. This perspective is particularly valuable for education research, as it captures early professional sense-making and exposes tensions between taught ideals and observed clinical practice, which may shape learners’ ethical development. The study provides insights into both the types of bias and discrimination encountered in clinical practice linked to specific patient characteristics and the situational progression of these incidents. The findings further suggest important implications for students’ future professional conduct, particularly with respect to developing ethical awareness and sensitivity to inequitable care. Although the study was conducted in a single institution, the results hold significance in health profession research by illuminating how learners identify and rationalise biased or discriminatory behaviour in clinical settings, serving as early indicator of professional norm negotiation. Student uncertainty and the limited visibility of institutional follow-up highlight system-level conditions that may either inhibit or enable ethical learning, speak-up behaviour, and future patient-facing professionalism. While these aspects strengthen the exploratory value of the study, they should be interpreted in light of the methodological constraints outlined below.

The study was conducted at a single university in the German-speaking part of Switzerland, with a predominance of female Swiss participants. This reflects the composition of healthcare students in Switzerland where the workforce is predominantly female. This composition may also have shaped the types of situations identified as discriminatory and the sensitivity to certain forms of bias, potentially underrepresenting perspectives related to other social or cultural positions. With respect to international settings, this, however, may limit generalisability or transferability. In particular, the findings may be transferable to healthcare education settings with comparable demographic compositions, training structures, and sociocultural contexts, while different patterns of observed discrimination may emerge in more heterogeneous or differently structured systems. Replicating the study in other regions, institutions, or more heterogeneous cohorts would strengthen understanding of transferability and expand the evidence base.

Many of the free-text responses were brief and lacked contextual detail to clearly distinguish between single observed incidents and general perception of biased and discriminatory behaviour, thereby limiting in-depth interpretation. This likely reflects the deliberately open wording of the question and its placement within a quantitative survey, a format which tends to elicit brief and unspecific responses, and may also relate to hurried or mobile-based survey completion.

While the absence of examples or prompts was an intentional design choice to avoid anchoring heuristics and to capture students’ own interpretations of observed biased or discriminatory behaviour, it may have contributed to variability in response depth and reduced the richness and specificity of some accounts. As a result, subtle, cumulative, or normalised forms of discriminatory practice may be underrepresented. Together with the relatively low response rate, it is therefore possible that certain types of observed discrimination were not fully captured. Alternative methods, such as focus groups or in-depth interviews, could complement the present approach and generate richer and more contextualised accounts.

In addition, the study did not capture students’ reflections on how biased or discriminatory behaviour affected them personally or shaped their professional development. Consequently, no conclusions can be drawn regarding processes of internalisation or the longer-term impact of these observations on professional socialisation. While such perspectives would be highly relevant for understanding processes of professional identity formation, they were beyond the scope of the present study. Future research using qualitative or longitudinal approaches could explore how these experiences are interpreted by students over time and how they influence professional values and conduct. Furthermore, future research should examine how institutional reporting structures, power hierarchies, and dependency relationships within healthcare systems shape students’ responses to observed or experienced discrimination. Future studies should also extend the perspective beyond students to include patients, relatives, and healthcare professionals themselves, in order to explore whether and how such behaviours are experienced, observed, or enacted across different stakeholder groups, and to better understand underlying mechanisms. Drawing on methodological approaches from research on racism and discrimination may further support the design of studies addressing these complex and sensitive phenomena. Finally, reliance on subjective, observer accounts entail inherent limitations, as all observations are shaped by perspective, and causal attribution (e.g., whether bias was conscious or unconscious) remains challenging. Students’ interpretations may be influenced by their level of training, prior experiences, and awareness of equity-related issues, which can shape both what is noticed and how behaviours are categorised, including the attribution of intent or severity.

## Conclusions

Healthcare students frequently witness biased or discriminatory behaviour towards patients, which is often linked to origin, language barriers, or certain diagnoses, and is typically displayed by senior professionals. Together with students’ reported uncertainty in identifying such behaviour and the limited visibility of the follow-up and consequences, these findings warrant attention, as repeated exposure to discriminatory behaviour may shape how learners interpret informal clinical and institutional norms.

The findings suggest that fostering a culture, in which equitable treatment is emphasized, discriminatory behaviour is openly addressed, and students are supported though structured opportunities for guided reflection may help align observed practice with formal educational goals. Providing clinical educators with training in recognising and addressing biases may further support this process. These considerations may be relevant not only in Switzerland but also in other healthcare systems facing increasingly diverse patient populations.

## Data Availability

The dataset generated and/or analysed during the current study is not publicly available due to data protection constraints. However, an anonymized dataset may be available from the corresponding author upon reasonable request, subject to verification of appropriate data handling in compliance with the GDPR.
